# The genetic susceptibility to type 2 diabetes may be modulated by obesity status: implications for association studies

**DOI:** 10.1186/1471-2350-9-45

**Published:** 2008-05-22

**Authors:** Stéphane Cauchi, Kevin T Nead, Hélène Choquet, Fritz Horber, Natascha Potoczna, Beverley Balkau, Michel Marre, Guillaume Charpentier, Philippe Froguel, David Meyre

**Affiliations:** 1 CNRS UMR8090, Institut de Biologie de Lille, Génomique et Physiologie Moléculaire des Maladies Métaboliques, Lille, France; 2 Department of Surgery and Internal Medicine, Hirslanden Clinics, Bern and Zurich, Switzerland; 3 INSERM U780-IFR69, Hôpital Paul Brousse, Villejuif, France; 4 Université Paris-Sud, Paris, France; 5 INSERM U695, Hôpital Bichat, Paris, France; 6 Hôpital de Corbeil, service d'endocrinologie et de diabétologie, Corbeil-Essonnes, France; 7 Genomic Medicine, Hammersmith Hospital, Imperial College London, UK

## Abstract

**Background:**

Considering that a portion of the heterogeneity amongst previous replication studies may be due to a variable proportion of obese subjects in case-control designs, we assessed the association of genetic variants with type 2 diabetes (T2D) in large groups of obese and non-obese subjects.

**Methods:**

We genotyped *RETN*, *KCNJ11*, *HNF4A*, *HNF1A*, *GCK*, *SLC30A8*, *ENPP1*, *ADIPOQ*, *PPARG*, and *TCF7L2 *polymorphisms in 1,283 normoglycemic (NG) and 1,581 T2D obese individuals as well as in 3,189 NG and 1,244 T2D non-obese subjects of European descent, allowing us to examine T2D risk over a wide range of BMI.

**Results:**

Amongst non-obese individuals, we observed significant T2D associations with *HNF1A *I27L [odds ratio (OR) = 1.14, *P *= 0.04], *GCK *-30G>A (OR = 1.23, *P *= 0.01), *SLC30A8 *R325W (OR = 0.87, *P *= 0.04), and *TCF7L2 *rs7903146 (OR = 1.89, *P *= 4.5 × 10^-23^), and non-significant associations with *PPARG *Pro12Ala (OR = 0.85, *P *= 0.14), *ADIPOQ *-11,377C>G (OR = 1.00, *P *= 0.97) and *ENPP1 *K121Q (OR = 0.99, *P *= 0.94). In obese subjects, associations with T2D were detected with *PPARG *Pro12Ala (OR = 0.73, *P *= 0.004), *ADIPOQ *-11,377C>G (OR = 1.26, *P *= 0.02), *ENPP1 *K121Q (OR = 1.30, *P *= 0.003) and *TCF7L2 *rs7903146 (OR = 1.30, *P *= 1.1 × 10^-4^), and non-significant associations with *HNF1A *I27L (OR = 0.96, *P *= 0.53), *GCK *-30G>A (OR = 1.15, *P *= 0.12) and *SLC30A8 *R325W (OR = 0.95, *P *= 0.44). However, a genotypic heterogeneity was only found for *TCF7L2 *rs7903146 (*P *= 3.2 × 10^-5^) and *ENPP1 *K121Q (*P *= 0.02). No association with T2D was found for *KCNJ11*, *RETN*, and *HNF4A *polymorphisms in non-obese or in obese individuals.

**Conclusion:**

Genetic variants modulating insulin action may have an increased effect on T2D susceptibility in the presence of obesity, whereas genetic variants acting on insulin secretion may have a greater impact on T2D susceptibility in non-obese individuals.

## Background

Type 2 diabetes (T2D) is characterized by defects in both insulin sensitivity and beta-cell dysfunction [1]. This disease involves a complex interaction between genetic variants and environment with obesity established as a primary risk factor [2]. In this regard, correlations between increased body fat and insulin resistance have been reliably demonstrated [3]. Recently, associations between polymorphisms and T2D were shown to be modulated according to obesity status.

*ENPP1*, *ADIPOQ*, *PPARG *and *TCF7L2 *single nucleotide polymorphisms (SNPs) have previously been associated with T2D [4-7] and have shown variable significance amongst different classes of obesity. It has been suggested that the *ENPP1 *K121Q and *ADIPOQ *-11,391G>A and -11,377C>G variants may confer greater T2D susceptibility in obese populations [8-10]. *PPARG *Pro12Ala, though controversial, was more associated with T2D amongst obese subgroups in our previous study [11-13]. Conversely, the *TCF7L2 *rs7903146 T allele has been shown to be more prevalent in T2D non-obese individuals compared to T2D obese subjects [7]. Interestingly, the variants in *PPARG*, *ADIPOQ*, and *ENPP1*, which elevate risk in obese populations, have been categorized as acting on insulin resistance while the *TCF7L2 *variant elevating risk in lean populations has been categorized as acting on insulin secretion [14-17]. These preliminary results suggest that different genetic architectures could increase T2D susceptibility according to the presence or absence of obesity.

Based on these observations, we have chosen to confirm previous findings and evaluate additional genetic variants which have a reported effect on either insulin action (*RETN *-420C>G) [18] or insulin secretion (*KCNJ11 *E23K, *HNF4A *rs1884614 and rs2144908, *HNF1A *I27L, *GCK *-30G>A, and *SLC30A8 *R325W) [19-25] and which have previously been associated with T2D in large scale studies or meta-analysis [21,26-30]. The evaluated variants were tested for their differential association with T2D in obese [1,283 normoglycemic (NG) and 1,581 T2D] and non-obese subjects (3,189 NG and 1,244 T2D) of European descent.

## Methods

### Study population

All NG and T2D subjects were recruited in three different centers, two in France and one in Switzerland. The clinical characteristics of the studied groups are presented in Table 1.

**Table 1:** Clinical characteristics and cohort distribution of the study population

**Obesity status**	**Glycemic status**	**Population**	**N**	**Sex ratio (men:women)**	**Age at examination (years)**	**BMI (kg/m^2^)**
**Non-obese**	**NG**	**French case-control group**	1,067	477:590	46 ± 15	24.3 ± 3.0
		**French D.E.S.I.R. cohort**	2,122	880:1,242	47 ± 10	22.7 ± 2.3
		**Swiss group**	na	na	na	na
		**Overall**	3,189	1357:1832	47 ± 12	23.3 ± 2.6
	**T2D**	**French case-control group**	1,244	805:439	60 ± 11	26.2 ± 2.6
		**French D.E.S.I.R. cohort**	na	na	na	na
		**Swiss group**	na	na	na	na
		**Overall**	1,244	805:439	60 ± 11	26.2 ± 2.6
**Obese**	**NG**	**French case-control group**	678	181:497	41 ± 11	41.0 ± 8.2
		**French D.E.S.I.R. cohort**	na	na	na	na
		**Swiss group**	605	128:477	42 ± 10	43.7 ± 7.0
		**Overall**	1,283	309:974	42 ± 11	42.2 ± 7.8
	**T2D**	**French case-control group**	1,347	645:702	56 ± 11	36.8 ± 7.1
		**French D.E.S.I.R. cohort**	na	na	na	na
		**Swiss group**	234	66:168	46 ± 11	42.7 ± 7.2
		**Overall**	1,581	711:870	54 ± 12	37.1 ± 7.4

*P*-values are from linear regression models adjusted for gender and age

NG: Normoglycemic

T2D: Type 2 Diabetic

na: Not available

Non-obese: BMI < 30 kg/m^2^

Obese: BMI ≥ 30 kg/m^2^

Age and BMI data presented as 'mean ± standard deviation'

Unrelated NG and T2D French white adults were recruited using a multimedia campaign run by the "Centre National de la Recherche Scientifique" (CNRS), the Department of Nutrition of the Paris Hôtel-Dieu Hospital and the Pasteur Institute of Lille.

Individuals both non-obese and NG (n = 2,122) of the French general population, aged between 30 and 65 years, participated in the cohort for the Data From an Epidemiological Study on the Insulin Resistance syndrome (D.E.S.I.R.), a 9-year follow-up study that aims to clarify the development of the insulin resistance syndrome and described previously [31]. Ethnic origin cannot be legally documented in France. We estimated the proportion of subjects having non-European ancestry from a subgroup of 654 subjects selected in the D.E.S.I.R. cohort, as previously described [32]. We genotyped 328 SNPs which were spaced by at least 5 Mb and highly differentiated among individuals from different continents (Fst > 0.2 based on the Perlegen dataset) [33]. Analysis using the STRUCTURE software identified only two individuals of non-european ancestry in a total of 654 individuals. From this analysis, the proportion of subjects having non-European ancestry was estimated to be 0.30% in the D.E.S.I.R. cohort. Additionally, all individuals born outside France were excluded from the study.

All NG and T2D Swiss white obese individuals were from Bern and Zurich as described previously [34]. They were added to the French samples of the same corpulence category. No heterogeneities were found between these two population groups for all SNPs [see Additional file 1].

In all centers, two classes of glycemic status were defined according to the 1997 American Diabetes Association criteria: NG, defined as fasting glucose < 6.1 mmol/l and T2D, defined as fasting plasma glucose ≥ 7.0 mmol/l. Two separate BMI categories were analyzed in this study, according to WHO international classification: non-obese (BMI < 30 kg/m^2^), and obese (BMI ≥ 30 kg/m^2^).

This genetic study was approved by the Ethical Committee of Hotel-Dieu in Corbeil-Essonnes and CHRU in Lille and informed consent was obtained from all participants.

### SNP genotyping

High-throughput genotyping of all genetic variants was performed using the TaqMan^® ^SNP Genotyping Assays (Applied Biosystems, Foster City, Calif. USA). The PCR primers and TaqMan probes were designed by Primer Express and optimized according to the manufacturer's protocol. There was a 96–99% genotyping success rate for all SNPs and the genotyping error rate was assessed by randomly genotyping 384 control and 384 T2D individuals. No difference was found from the first genotyping results, thus the genotyping error rate was 0% for each SNP. The genotypic distributions for all SNPs were in Hardy-Weinberg equilibrium (P ≥ 0.01) [see Additional file 2].

### Statistical analysis

The association with T2D was calculated using a logistic regression model adjusted for age, gender, and BMI. The minimum detectable effect size with a statistical power of 80% was assessed using Quanto [see Additional file 3]. The Woolf test was applied to asses the genotypic heterogeneity between obese and non-obese groups. No Bonferroni correction was applied as, in the case of replication studies, it is unlikely to detect effects due to statistical fluctuation only. All *P *values are two-sided. SPSS (version 14.0.2) and R statistics (version 2.5.0) software were used for general statistics.

## Results

All SNPs were evaluated for their contribution to T2D in the complete sample population (4,472 NG and 2,825 T2D), then in obese individuals (1,283 NG and 1,581 T2D) and non-obese subjects (3,189 NG and 1,244 T2D). Clinical characteristics and allelic distributions of the study population are summarized in Table 1 and Table 2, respectively. The best fitting genetic models were selected from previous studies [5-7,13,26-30,35] and were in agreement with what we found in our analyses (data not shown).

**Table 2:** Genotypic distributions in obese and non-obese subjects by glycemic status

		**Non-obese**						**Obese**					
		
**Gene**	**SNP**	**Genotype 1-1**		**Genotype 1-2**		**Genotype 2-2**		**Genotype 1-1**		**Genotype 1-2**		**Genotype 2-2**	
		**T2D**	**NG**	**T2D**	**NG**	**T2D**	**NG**	**T2D**	**NG**	**T2D**	**NG**	**T2D**	**NG**
*ADIPOQ*	rs17300539	**0.82 **_(1,011)_	**0.82 **_(2,579)_	**0.16 **_(199)_	**0.17 **_(541)_	**0.01 **_(16)_	**0.01 **_(37)_	**0.81 **_(1238)_	**0.84 **_(1059)_	**0.18 **_(274)_	**0.15 **_(189)_	**0.01 **_(15)_	**0.01 **_(15)_
*ADIPOQ*	rs266729	**0.56 **_(682)_	**0.56 **_(1,753)_	**0.38 **_(461)_	**0.37 **_(1164)_	**0.07 **_(83)_	**0.07 **_(228)_	**0.55 **_(860)_	**0.6 **_(761)_	**0.39 **_(611)_	**0.33 **_(425)_	**0.06 **_(89)_	**0.07 **_(85)_
*ENPP1*	rs1044498	**0.73 **_(893)_	**0.72 **_(2,227)_	**0.25 **_(308)_	**0.25 **_(785)_	**0.02 **_(29)_	**0.03 **_(79)_	**0.7 **_(1086)_	**0.74 **_(917)_	**0.27 **_(414)_	**0.23 **_(286)_	**0.03 **_(53)_	**0.03 **_(32)_
*PPARG*	rs1801282	**0.82 **_(1010)_	**0.79 **_(2498)_	**0.17 **_(209)_	**0.2 **_(638)_	**0.01 **_(9)_	**0.01 **_(30)_	**0.81 **_(1232)_	**0.76 **_(920)_	**0.18 **_(270)_	**0.22 **_(270)_	**0.01 **_(19)_	**0.02 **_(20)_
*RETN*	rs1862513	**0.48 **_(590)_	**0.49 **_(1499)_	**0.43 **_(533)_	**0.41 **_(1249)_	**0.09 **_(107)_	**0.1 **_(291)_	**0.5 **_(775)_	**0.48 **_(572)_	**0.4 **_(626)_	**0.44 **_(530)_	**0.1 **_(150)_	**0.08 **_(102)_
*GCK*	rs1799884	**0.64 **_(747)_	**0.68 **_(2045)_	**0.32 **_(369)_	**0.29 **_(858)_	**0.04 **_(48)_	**0.03 **_(97)_	**0.65 **_(960)_	**0.67 **_(775)_	**0.32 **_(466)_	**0.3 **_(347)_	**0.03 **_(47)_	**0.03 **_(37)_
*HNF1A*	rs1169288	**0.44 **_(532)_	**0.47 **_(1373)_	**0.44 **_(539)_	**0.43 **_(1270)_	**0.12 **_(151)_	**0.1 **_(295)_	**0.47 **_(718)_	**0.44 **_(537)_	**0.43 **_(658)_	**0.44 **_(538)_	**0.11 **_(168)_	**0.12 **_(143)_
*HNF4A*	rs1884614	**0.68 **_(840)_	**0.69 **_(2163)_	**0.29 **_(354)_	**0.28 **_(873)_	**0.03 **_(35)_	**0.03 **_(87)_	**0.7 **_(1078)_	**0.69 **_(825)_	**0.28 **_(429)_	**0.28 **_(340)_	**0.03 **_(44)_	**0.03 **_(31)_
*HNF4A*	rs2144908	**0.68 **_(814)_	**0.69 **_(2153)_	**0.3 **_(355)_	**0.28 **_(873)_	**0.03 **_(34)_	**0.03 **_(91)_	**0.69 **_(1051)_	**0.68 **_(811)_	**0.28 **_(429)_	**0.29 **_(348)_	**0.03 **_(43)_	**0.03 **_(31)_
*KCNJ11*	rs5219	**0.41 **_(489)_	**0.38 **_(1145)_	**0.45 **_(537)_	**0.48 **_(1438)_	**0.15 **_(180)_	**0.15 **_(439)_	**0.41 **_(623)_	**0.4 **_(480)_	**0.45 **_(683)_	**0.47 **_(568)_	**0.15 **_(222)_	**0.14 **_(164)_
*SLC30A8*	rs13266634	**0.55 **_(661)_	**0.49 **_(1497)_	**0.38 **_(455)_	**0.42 **_(1264)_	**0.08 **_(94)_	**0.09 **_(275)_	**0.53 **_(792)_	**0.51 **_(617)_	**0.4 **_(599)_	**0.42 **_(509)_	**0.08 **_(114)_	**0.08 **_(93)_
*TCF7L2*	rs7903146	**0.3 **_(366)_	**0.48 **_(1,512)_	**0.5 **_(607)_	**0.42 **_(1326)_	**0.2 **_(243)_	**0.09 **_(290)_	**0.41 **_(626)_	**0.5 **_(614)_	**0.46 **_(715)_	**0.41 **_(502)_	**0.13 **_(203)_	**0.1 **_(119)_

Data presented as Frequency (n)

T2D: Type 2 diabetic

NG: Normoglycemic

Non-obese: BMI < 30 kg/m^2^

Obese: BMI ≥ 30 kg/m^2^

1: Major allele

2: Minor allele

### All NG and T2D Individuals

We first evaluated the genetic variants amongst all NG and T2D individuals regardless of obesity status (Table 3). As previously reported in a more modestly-sized French set, T2D associations were confirmed in *PPARG *Pro12Ala [odds ratio (OR) = 0.81, *P *= 0.004], *TCF7L2 *rs7903146 (OR = 1.59, *P *= 7.9 × 10^-27^), and *ENPP1 *K121Q (OR = 1.15, *P *= 0.01) [7-9,11]. Additionally, *GCK *-30G>A (OR 1.20, *P *= 0.001) and *SLC30A8 *R325W (OR = 0.91, *P *= 0.03) were also found to increase T2D risk.

**Table 3:** Type 2 diabetes case-control study by obesity status

**Functional relevance**	**Gene name**	**SNP rs ID**	**Genetic model**	**Obesity status**	**OR [95%CI]**	***P *value**	**Woolf test**	**Risk allele frequency**	
								**T2D**	**NG**

**Insulin action**	***ADIPOQ***	rs17300539	Dominant	Non-obese	0.96 [0.77–1.20]	0.71	0.5	0.09	0.10
				Obese	1.07 [0.84–1.35]	0.58		0.10	0.09
				All together	0.99 [0.86–1.15]	0.91		0.10	0.09
	***ADIPOQ***	rs266729	Dominant	Non-obese	1.00 [0.85–1.19]	0.97	0.07	0.26	0.26
				Obese	**1.25 [1.04–1.49]**	**0.015**		0.25	0.24
				All together	1.08 [0.96–1.21]	0.18		0.25	0.25
	***ENPP1***	rs1044498	Additive	Non-obese	0.99 [0.84–1.17]	0.94	**0.02**	0.15	0.15
				Obese	**1.30 [1.10–1.54]**	**0.003**		0.17	0.14
				All together	**1.15 [1.03–1.28]**	**0.014**		0.16	0.15
	***PPARG***	rs1801282	Recessive	Non-obese	0.85 [0.69–1.05]	0.136	0.24	0.91	0.89
				Obese	**0.73 [0.58–0.90]**	**0.004**		0.90	0.87
				All together	**0.81 [0.71–0.94]**	**0.004**		0.90	0.89
	***RETN***	rs1862513	Recessive	Non-obese	0.81 [0.60–1.09]	0.16	0.11	0.30	0.30
				Obese	1.14 [0.84–1.55]	0.42		0.30	0.31
				All together	1.00 [0.82–1.22]	0.99		0.30	0.30
**Beta-cell defect**	***GCK***	rs1799884	Additive	Non-obese	**1.23 [1.06–1.45]**	**0.009**	0.55	0.20	0.17
				Obese	1.15 [0.97–1.35]	0.12		0.19	0.18
				All together	**1.20 [1.08–1.34]**	**0.001**		0.19	0.18
	***HNF1A***	rs1169288	Additive	Non-obese	**1.14 [1.01–1.30]**	**0.042**	0.06	0.34	0.32
				Obese	0.96 [0.84–1.10]	0.53		0.32	0.34
				All together	1.02 [0.93–1.11]	0.71		0.33	0.32
	***HNF4A***	rs1884614	Additive	Non-obese	1.06 [0.90–1.24]	0.51	0.94	0.17	0.17
				Obese	1.05 [0.89–1.25]	0.54		0.17	0.17
				All together	1.03 [0.93–1.15]	0.58		0.17	0.17
	***HNF4A***	rs2144908	Additive	Non-obese	1.08 [0.92–1.27]	0.33	0.75	0.18	0.17
				Obese	1.04 [0.88–1.24]	0.63		0.17	0.17
				All together	1.04 [0.94–1.16]	0.45		0.17	0.17
	***KCNJ11***	rs5219	Dominant	Non-obese	0.93 [0.78–1.10]	0.38	0.8	0.37	0.38
				Obese	0.96 [0.80–1.15]	0.68		0.37	0.37
				All together	0.90 [0.80–1.01]	0.085		0.37	0.38
	***SLC30A8***	rs13266634	Additive	Non-obese	**0.87 [0.76–0.99]**	**0.043**	0.37	0.73	0.70
				Obese	0.95 [0.82–1.09]	0.44		0.73	0.71
				All together	**0.91 [0.83–0.99]**	**0.033**		0.73	0.70
	***TCF7L2***	rs7903146	Additive	Non-obese	**1.89 [1.67–2.14]**	**4.5 × 10**^-23^	**3.2 × 10**^-5^	0.45	0.30
				Obese	**1.30 [1.14–1.48]**	**1.1 × 10**^-4^		0.36	0.30
				All together	**1.59 [1.46–1.73]**	**7.9 × 10**^-27^		0.40	0.30

Odds ratios were adjusted for age, gender and BMI

Woolf test: Test for heterogeneity in genotypic distribution between obese and non-obese subjects

Non-obese: BMI < 30 kg/m^2^

Obese: BMI ≥ 30 kg/m^2^

In contrast, in our whole sample analysis, no evidence for association with T2D was found for the *ADIPOQ *variants -11,391G>A (OR = 0.99, *P *= 0.91) and -11,377C>G (OR = 1.08, *P *= 0.18), *HNF1A *I27L (OR = 1.01, *P *= 0.71), *HNF4A *variants rs1884614 (OR = 1.03, *P *= 0.58) and rs2144908 (OR = 1.04, *P *= 0.45), *KCNJ11 *E23K (OR = 0.9, *P *= 0.09), or *RETN *-420C>G (OR = 1.00, *P *= 0.99).

### Non-Obese T2D Association

We then assessed the genetic variants amongst the non-obese population (BMI < 30 kg/m^2^) (Table 3). The risk alleles of *HNF1A *I27L (OR = 1.14, *P *= 0.04), *GCK *-30G>A (OR = 1.23, *P *= 0.01), *SLC30A8 *R325W (OR = 0.87, *P *= 0.04), and *TCF7L2 *rs7903146 (OR = 1.89, *P *= 4.5 × 10^-23^) conferred a significantly increased risk of T2D.

The *ADIPOQ *variants -11,391G>A (OR = 0.96, *P *= 0.71) and -11,377C>G (OR = 1.00, *P *= 0.97), *ENPP1 *K121Q (OR = 0.99, *P *= 0.94), *PPARG *Pro12Ala (OR = 0.85, *P *= 0.14), *RETN *-420C>G (OR = 0.41, *P *= 0.14), *KCNJ11 *E23K (OR = 0.96, *P *= 0.68), and *HNF4A *variants rs1884614 (OR = 1.05, *P *= 0.54) and rs2144908 (OR = 1.08, *P *= 0.33) were not associated with T2D amongst the non-obese group.

### Obese T2D Association

In the obese group (BMI ≥ 30 kg/m^2^), associations with T2D were detected in *PPARG *Pro12Ala (OR 0.73, *P *= 0.004), *ADIPOQ *-11,377C>G (OR = 1.25, *P *= 0.015), *ENPP1 *K121Q (OR = 1.30, *P *= 0.003) and *TCF7L2 *rs7903146 (OR = 1.30, *P *= 1.1 × 10^-4^) (Table 3).

We did not find evidence for increased T2D risk for *GCK *-30G>A (OR = 1.15, *P *= 0.12), *ADIPOQ *-11,391G>A (OR = 1.07, *P *= 0.58), *HNF1A *I27L (OR = 0.96, *P *= 0.53), *HNF4A *variants rs1884614 (OR = 1.05, *P *= 0.54) and rs2144908 (OR = 1.04, *P *= 0.63), *KCNJ11 *E23K (OR = 0.96, *P *= 0.68), or *SLC30A8 *R325W (OR = 0.95, *P *= 0.44) amongst obese subjects.

### Genotypic heterogeneity

Discrepancies in genotypic distribution were assessed by Woolf test between the obese and non-obese categories (Table 3). A genotypic heterogeneity was found for *TCF7L2 *rs7903146 (*P *= 3.2 × 10^-5^) and *ENPP1 *K121Q (*P *= 0.02). Their association with BMI change was then assessed in all studied subgroups. Significant effects on BMI were only detected in the T2D group for the *TCF7L2 *genetic variant [see Additional file 4]. Trends towards heterogeneity were found for *ADIPOQ *11377C>G (*P *= 0.07) and *HNF1A *I27L (*P *= 0.06).

## Discussion

This large case-control study further supports the hypothesis that the T2D risk contribution of *ENPP1*, *ADIPOQ*, *PPARG *and *TCF7L2 *SNPs may be modulated by obesity status [6-8,11]. Notably, we made the novel finding that T2D risk, as conferred by *HNF1A*, *GCK*, and *SLC30A8 *genetic variants, may also be modified in the presence/absence of obesity. Despite the stringent standards exacted by the Woolf test, genotypic heterogeneity between obese and non-obese individuals was detected for *TCF7L2 *rs7903146 and *ENPP1 *K121Q, and suggested for *ADIPOQ *11377C>G and *HNF1A *I27L SNPs. Future study designs containing more individuals will be needed to confirm all apparent genotypic heterogeneities.

The analysis of *HNF4A*, *KCNJ11 *and *RETN *polymorphisms did not support their role as T2D risk factors despite sufficient statistical power [see Additional file 3] to detect the effect sizes (1.08, 1.09 and 1.16, respectively) previously established by meta-analysis [21,26,27]. The inability of the current study to replicate previous findings is likely the result of true but modest contributions to T2D risk in the European population.

While it is known that defects in insulin action and insulin secretion are critical in T2D pathogenesis, their interaction with genetic and environmental factors is less clear. Interestingly, *SLC30A8*, *GCK*, and *HNF1A *genetic variants, all of which have been associated with insulin secretion [22-25], were only associated with T2D in non-obese individuals. The *TCF7L2 *genetic variant, another SNP modulating beta cell function [17,36], was more associated with T2D in non-obese subjects than in obese individuals. Conversely, insulin resistance associated genetic variants of *PPARG*, *ADIPOQ *and *ENPP1 *[14-16,37,38] were found to be only associated in obese subjects. While obesity has been strongly associated with increased insulin resistance [3], our data suggests that insulin secretory variants confer a greater T2D risk in non-obese individuals while insulin sensitivity variants more significantly modulate T2D risk in obese subjects.

Currently, some dissension exists regarding the BMI modulation of the Pro12Ala SNP in T2D risk. Contrary to what was reported by Ghoussaini and colleagues [11], other studies of the *PPARG *Pro12Ala SNP suggest that the protective effect of the Ala allele may be greater when control BMI is lower [12,13]. The present analysis was specifically designed to detect interactions by analyzing a large number of European subjects with no genotypic heterogeneity and characterized by a wide range of BMI. In the "Diabetes Prevention Program", 55 percent of participants were Caucasian, 45 percent were minorities, most were obese, and most had a family history of T2D [12]. Similarly, Ludivico and colleagues performed a combined analysis of Asian, North American, and European populations. Interestingly, the genetic effect on T2D was found to be ~30% stronger in Asians than in the two other more corpulent populations [13]. We therefore suggest that population heterogeneity may be the primary contributor to the overall observed BMI effect as supported by the ability of BMI to statistically explain the heterogeneity between these populations but not within Europeans [13]. Large ethnically-matched studies would be necessary to know if such interaction is found in non-European subjects.

These results illustrate the importance of BMI in population design as obesity mediated T2D associations may account for part of the difficulty encountered in replication studies. The *ENPP1 *K121Q variant may be illustrative of this point. Studies exploring the T2D risk contribution of the K121Q SNP amongst obese individuals [8,10] have found an enhanced association in this subgroup. In the present study, we supported these results in finding no significant effect on T2D in non-obese individuals (OR = 0.99, *P *= 0.94), an association with T2D in obese subjects (OR = 1.30, *P *= 0.003) and a subsequent modest effect when obese and non-obese samples were analyzed together (OR = 1.15, *P *= 0.01). A portion of the heterogeneity amongst previous replication studies may be due to a variable fraction of obese subjects in study designs [5,8,39,40]. Considering these results, future association studies should take into account obesity status when interpreting their data.

**Figure 1:**
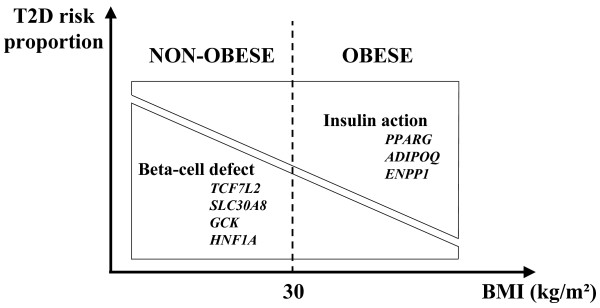
**Functional relevance of genetic variants may affect their association with T2D by obesity status**. The present figure does not take into account variants associated with T2D through their effect on BMI.

A representation of the possible relationship between T2D risk variants and obesity status is modeled in Figure 1. This study suggests that the magnitude of a variant's effect on T2D susceptibility is modulated by both obesity status and SNP function. Specifically, variants effecting insulin action more significantly increase T2D susceptibility in obese individuals while variants effecting insulin secretion confer greater T2D risk in non-obese individuals. In our study, data on waist/hip ratio were not available in most T2D cases. However, further analyses of the issue should consider this anthropometric parameter when analyzing interactions between obesity and T2D.

These data are concordant with results from previous epidemiological and physiological studies. Prospective data indicates that impaired beta cell function, not insulin resistance, predicts future T2D in non-obese subjects [41]. Furthermore, it was recently reported that interactions between *TCF7L2 *genetic variants and adiposity may lead to reduced beta-cell compensation in leaner individuals but facilitate improved compensation in more obese subjects [42]. Increased beta-cell mass has indeed been observed in the pancreata of obese compared to lean non-diabetic subjects [43]. These results support a primary role of insulin secretory modulating variants in the absence of obesity. Conversely, the hyperbolic relationship of insulin resistance and insulin secretion enables the beta-cell to adequately maintain glucose homeostasis for a large range of insulin resistance levels [44]. In non-obese subjects not affected by adiposity-induced insulin resistance, this mechanism may sufficiently compensate the effects of genetic variants increasing insulin resistance.

Obesity-induced insulin resistance is characterized by a series of events: i) impaired regulation of lipolysis in adipocytes, ii) increased circulating levels of free fatty acids [45], iii) ectopic fat storage in muscle and liver, iv) impaired ability of insulin to inhibit hepatic glucose production and muscular glucose uptake [46], v) increased beta cell death by apoptosis (glucolipotoxicity) [43,47]. In obesity-prone populations, insulin resistance more accurately predicts development of T2D than does insulin secretory dysfunction [48]. Previous studies on lipodistrophy in both human and murine models have shown that adipose tissue plays a key role in insulin resistance [49]. Interestingly, *PPARG*, *ADIPOQ*, and *ENPP1 *SNPs only associated with T2D in obese subjects, and have a pivotal role in adipocyte differentiation, maturation, and action [50-52]. The effects of genetic polymorphisms leading to insulin resistance may therefore be worsened by obesity.

In the present study, we suggested that the genetic architecture of T2D may be different in obese and non-obese individuals. In a given ethnic group, the lack of replication in association studies may be, in part, due to different fractions of obesity in case-control designs. Novel SNPs recently identified in genome-wide association studies will prove important in the confirmation of these findings once their functions have been reliably demonstrated.

## Abbreviations

T2D: Type 2 Diabetes; NG: Normoglycemic; BMI: Body Mass Index; OR: Odds ratio; SNP: Single Nucleotide Polymorphism.

## Competing interests

The authors declare that they have no competing interests.

## Authors' contributions

SC managed the study, carried out the genetic analyses and drafted the manuscript. KTN carried out the genetic analyses, drafted the manuscript and carried out the genotyping experiments. HC carried out the genotyping experiments. FH participated in the design of the study. NP participated in the design of the database. BB participated in the design of the study. MM participated in the design of the study. GC participated in the design of the study. PF coordinated the study. DM conceived the study, and participated in its design and coordination. All authors read and approved the final manuscript.

## Pre-publication history

The pre-publication history for this paper can be accessed here:



## Supplementary Material

Additional file 1 Supplementary table 1. Homogeneity in genotypic distributions between Swiss and French subjects Click here for file

Additional file 2 Supplementary table 2. Hardy-Weinberg equilibrium for each studied SNP Click here for file

Additional file 3 Supplementary table 3. Minimum detectable effect size with a statistical power of 80%. Click here for file

Additional file 4 Supplementary table 4. BMI distribution by *ENPP1 *and *TCF7L2 *genotypes. Click here for file
