# Supplementary material for: The genetic susceptibility to type 2 diabetes may be modulated by obesity status: implications for association studies
Source: BMC Med Genet. 2008 May 22;9:45. doi: 10.1186/1471-2350-9-45 (PMC2412856; doi:10.1186/1471-2350-9-45)
Supplement: Additional file 1 — Supplementary table 1. Homogeneity in genotypic distributions between Swiss and French subjects [file 1471-2350-9-45-S1.doc]

**Supplementary Table 1**

**Homogeneity in genotypic distributions between Swiss and French subjects**

| **Gene** | **SNP** | **OR [95 % CI]** | | **Woolf** |
| --- | --- | --- | --- | --- |
| **name** | **rs ID** | **Swiss** | **French** | **test** |
| ***ADIPOQ*** | rs17300539 | 1.17 [0.75-1.82] | 0.89 [0.66-1.18] | 0.30 |
| ***ADIPOQ*** | rs266729 | 1.29 [0.94-1.77] | 1.28 [1.02-1.60] | 0.97 |
| ***ENPP1*** | rs1044498 | 1.10 [0.81-1.51] | 1.34 [1.08-1.66] | 0.30 |
| ***GCK*** | rs1799884 | 1.08 [0.80-1.46] | 1.21 [0.98-1.50] | 0.54 |
| ***HNF1A*** | rs1169288 | 0.90 [0.71-1.15] | 0.99 [0.83-1.16] | 0.52 |
| ***HNF4A*** | rs1884614 | 1.01 [0.74-1.37] | 1.07 [0.86-1.32] | 0.76 |
| ***HNF4A*** | rs2144908 | 0.98 [0.72-1.34] | 1.05 [0.85-1.30] | 0.71 |
| ***KCNJ11*** | rs5219 | 0.84 [0.61-1.17] | 0.98 [0.78-1.23] | 0.44 |
| ***PPARG*** | rs1801282 | 0.68 [0.45-1.03] | 0.73 [0.56-0.96] | 0.77 |
| ***RETN*** | rs1862513 | 1.48 [0.82-2.69] | 0.98 [0.68-1.42] | 0.24 |
| ***SLC30A8*** | rs13266634 | 0.98 [0.71-1.35] | 0.85 [0.68-1.07] | 0.47 |
| ***TCF7L2*** | rs7903146 | 1.05 [0.83-1.34] | 1.37 [1.16-1.62] | 0.07 |

Woolf test: Test for heterogeneity in genotypic distribution
