# Supplementary material for: The genetic susceptibility to type 2 diabetes may be modulated by obesity status: implications for association studies
Source: BMC Med Genet. 2008 May 22;9:45. doi: 10.1186/1471-2350-9-45 (PMC2412856; doi:10.1186/1471-2350-9-45)
Supplement: Additional file 2 — Supplementary table 2. Hardy-Weinberg equilibrium for each studied SNP [file 1471-2350-9-45-S2.doc]

**Supplementary Table 2**

**Hardy-Weinberg equilibrium for each studied SNP**

| **Gene** | **SNP** | **Hardy-Weinberg equilibrium (*P* values)** | |
| --- | --- | --- | --- |
| **name** | **rs ID** | **NG** | **T2D** |
| ***ADIPOQ*** | rs17300539 | 0.02 | 0.29 |
| ***ADIPOQ*** | rs266729 | 0.01 | 0.43 |
| ***ENPP1*** | rs1044498 | 0.05 | 0.11 |
| ***GCK*** | rs1799884 | 0.72 | 0.57 |
| ***HNF1A*** | rs1169288 | 0.76 | 0.21 |
| ***HNF4A*** | rs1884614 | 0.78 | 0.93 |
| ***HNF4A*** | rs2144908 | 0.88 | 0.62 |
| ***KCNJ11*** | rs5219 | 0.77 | 0.03 |
| ***PPARG*** | rs1801282 | 0.14 | 0.67 |
| ***RETN*** | rs1862513 | 0.41 | 0.63 |
| ***SLC30A8*** | rs13266634 | 0.93 | 0.38 |
| ***TCF7L2*** | rs7903146 | 0.57 | 0.87 |

T2D: Type 2 diabetic

NG: Normoglycemic
