# Supplementary material for: The genetic susceptibility to type 2 diabetes may be modulated by obesity status: implications for association studies
Source: BMC Med Genet. 2008 May 22;9:45. doi: 10.1186/1471-2350-9-45 (PMC2412856; doi:10.1186/1471-2350-9-45)
Supplement: Additional file 3 — Supplementary table 3. Minimum detectable effect size with a statistical power of 80%. [file 1471-2350-9-45-S3.doc]

**Supplementary Table 3**

**Minimum detectable effect size with a statistical power of 80%**

| **Gene** | **SNP** | **Best** | **Odds ratio** | | |
| --- | --- | --- | --- | --- | --- |
| **name** | **rs ID** | **Model** | **Non-obese** | **Obese** | **All together** |
| *ADIPOQ* | rs17300539 | Dominant | 1.13 | 1.22 | 1.11 |
| *ADIPOQ* | rs266729 | Dominant | 1.11 | 1.17 | 1.09 |
| *ENPP1* | rs1044498 | Additive | 1.12 | 1.16 | 1.10 |
| *PPARG* | rs1801282 | Recessive | 0.89 | 0.82 | 0.90 |
| *RETN* | rs1862513 | Recessive | 1.19 | 1.31 | 1.16 |
| *GCK* | rs1799884 | Additive | 1.10 | 1.16 | 1.08 |
| *HNF1A* | rs1169288 | Additive | 1.08 | 1.13 | 1.07 |
| *HNF4A* | rs1884614 | Additive | 1.10 | 1.16 | 1.08 |
| *HNF4A* | rs2144908 | Additive | 1.10 | 1.16 | 1.08 |
| *KCNJ11* | rs5219 | Dominant | 1.11 | 1.18 | 1.09 |
| *SLC30A8* | rs13266634 | Recessive | 0.92 | 0.88 | 0.94 |
| *TCF7L2* | rs7903146 | Additive | 1.08 | 1.13 | 1.07 |

Non-obese: BMI < 30kg/m²

Obese: BMI ≥ 30kg/m²
