# Supplementary material for: The genetic susceptibility to type 2 diabetes may be modulated by obesity status: implications for association studies
Source: BMC Med Genet. 2008 May 22;9:45. doi: 10.1186/1471-2350-9-45 (PMC2412856; doi:10.1186/1471-2350-9-45)
Supplement: Additional file 4 — Supplementary table 4. BMI distribution by ENPP1 and TCF7L2 genotypes. [file 1471-2350-9-45-S4.doc]

**Supplementary Table 4**

**BMI distribution by *ENPP1* and *TCF7L2* genotypes**

| **Gene** | **SNP** | **Glycemic** | **Obesity** | **Body Mass Index (kg/m²)** | | | ***P*** |
| --- | --- | --- | --- | --- | --- | --- | --- |
| **name** | **rs ID** | **status** | **status** | **1-1** | **1-2** | **2-2** | **value** |
| ***ENPP1*** | **rs1044498** | **NG** | **Non-Obese** | 23.25 ± 2.66 | 23.22 ± 2.60 | 23.65 ± 2.40 | 0.75 |
|  |  |  | **Obese** | 42.36 ± 7.94 | 41.6 ± 7.20 | 42.83 ± 8.44 | 0.45 |
|  |  |  | **All together** | 28.83 ± 9.94 | 28.12 ± 9.22 | 29.18 ± 10.02 | 0.20 |
|  |  | **T2D** | **Non-Obese** | 26.14 ± 2.57 | 26.19 ± 2.60 | 26.03 ± 2.71 | 0.76 |
|  |  |  | **Obese** | 37.74 ± 7.60 | 37.25 ± 6.60 | 40.01 ± 8.16 | 0.68 |
|  |  |  | **All together** | 32.5 ± 8.25 | 32.53 ± 7.60 | 35.06 ± 9.51 | 0.81 |
| ***TCF7L2*** | **rs7903146** | **NG** | **Non-Obese** | 23.29 ± 2.63 | 23.25 ± 2.70 | 23.31 ± 2.56 | 0.74 |
|  |  |  | **Obese** | 42.4 ± 7.90 | 41.93 ± 7.71 | 42.34 ± 8.00 | 0.48 |
|  |  |  | **All together** | 28.81 ± 9.90 | 28.38 ± 9.55 | 28.84 ± 9.90 | 0.50 |
|  |  | **T2D** | **Non-Obese** | 26.26 ± 2.62 | 26.08 ± 2.64 | 26.15 ± 2.42 | 0.62 |
|  |  |  | **Obese** | 37.99 ± 7.35 | 37.79 ± 7.67 | 36.51 ± 6.41 | **0.009** |
|  |  |  | **All together** | 33.66 ± 8.29 | 32.42 ± 8.31 | 30.87 ± 6.96 | **2.02x10^-10^** |

*P*-values are from linear regression models adjusted for gender and age

T2D: Type 2 Diabetic

Non-obese: BMI < 30kg/m²

Obese: BMI ≥ 30kg/m²

1: Major allele

2: Minor allele

BMI data presented as 'mean ± standard deviation'
